# Supplementary material for: The Systems Biology Research Tool: evolvable open-source software
Source: BMC Syst Biol. 2008 Jun 29;2:55. doi: 10.1186/1752-0509-2-55 (PMC2446383; doi:10.1186/1752-0509-2-55)
Supplement: Additional file 1 — SBRT Archive. An archive of the current version of the Systems Biology Research Tool. [file 1752-0509-2-55-S1.zip › sbrt-1.4.0/doc/developers_guide/api/sbrt/shell/io/AbstractMapInputFile.html]

AbstractMapInputFile


|  |  |  |
| --- | --- | --- |
| **Overview** \| **Package** \| **Class** \| **Use** \| **Tree** \| **Deprecated** \| **Index** \| **Help** | |  |
| PREV CLASS   **NEXT CLASS** | **FRAMES**    **NO FRAMES**     **All Classes** |  |
| SUMMARY: NESTED \| FIELD \| CONSTR \| METHOD | DETAIL: FIELD \| CONSTR \| METHOD |  |


---


## sbrt.shell.io Class AbstractMapInputFile<K,V>

```
java.lang.Object
  sbrt.shell.io.AbstractMapInputFile<K,V>
```

**Type Parameters:**: `K` - the key type.: `V` - the value type.

**All Implemented Interfaces:**: java.lang.Iterable<java.util.Map<K,V>>, InputFile<java.util.Map<K,V>>, MapInputFile<K,V>, MapTextFile<K,V>, MapTextInputFile<K,V>, SbrtFile

**Direct Known Subclasses:**: ComiFile, RomiFile

---

``` public abstract class AbstractMapInputFile<K,V> extends java.lang.Object implements MapTextInputFile<K,V> ```

This abstract class is a skeleton implemenation of text-based
map input files.

**Author:**
:   This class was written and documented by
    Jeremiah Wright while in the Wagner lab.

---

**Constructor Summary**

`AbstractMapInputFile(java.lang.String fileName,
FileFormat fileFormat,
MapFormat<K,V> mapFormat)`
  
          Constructs a new map input file from the provided arguments.

`AbstractMapInputFile(java.lang.String fileName,
FileFormat fileFormat,
java.util.Set<? extends K> requiredKeys,
MapFormat<K,V> mapFormat)`
  
          Constructs a new map input file from the provided arguments.


| **Method Summary** | |
| --- | --- |
| `void` | `close()`             Closes this file. |
| `java.lang.String` | `getCanonicalName()`             Returns the canonical name of this file. |
| `MapFormat<K,V>` | `getMapFormat()`             Returns the map format used to parse this file. |
| `java.lang.String` | `getOriginalName()`             Returns the name of this file. |
| `UnmodifiableSet<K>` | `getRequiredKeys()`             Returns the set of keys that must be contained in this file. |
| `boolean` | `isClosed()`             Indicates if this file has already been closed. |
| `protected  TextFileLineIterator<java.lang.String>` | `lineIterator()`             Returns a plain text line iterator over this text file. |

**Methods inherited from class java.lang.Object**

`clone, equals, finalize, getClass, hashCode, notify, notifyAll, toString, wait, wait, wait`

**Methods inherited from interface java.lang.Iterable**

`iterator`

**Constructor Detail**

### AbstractMapInputFile

```
public AbstractMapInputFile(java.lang.String fileName,
                            FileFormat fileFormat,
                            java.util.Set<? extends K> requiredKeys,
                            MapFormat<K,V> mapFormat)
```

:   Constructs a new map input file from the provided arguments.

    **Parameters:**: `fileName` - the name of the file to read.: `fileFormat` - the format of the file.: `requiredKeys` - the set of keys this file must contain.: `mapFormat` - the map format used to parse this file.

---


### AbstractMapInputFile

```
public AbstractMapInputFile(java.lang.String fileName,
                            FileFormat fileFormat,
                            MapFormat<K,V> mapFormat)
```

:   Constructs a new map input file from the provided arguments.

    **Parameters:**: `fileName` - the name of the file to read.: `fileFormat` - the format of the file.: `mapFormat` - the map format used to parse this file.


**Method Detail**

### getRequiredKeys

```
public UnmodifiableSet<K> getRequiredKeys()
```

:   Returns the set of keys that must be contained in this
    file.

    :   **Returns:**: the set of keys that must be contained in this file.

---


### getMapFormat

```
public MapFormat<K,V> getMapFormat()
```

:   Returns the map format used to parse this file.

    :   **Specified by:**: `getMapFormat` in interface `MapTextFile<K,V>`
    :   **Returns:**: the map format used to parse this file.

---


### lineIterator

```
protected final TextFileLineIterator<java.lang.String> lineIterator()
```

:   Returns a plain text line iterator over this text file.

    :   **Returns:**: a plain text line iterator over this text file.

---


### close

```
public void close()
```

:   Closes this file. If this file is already closed, then
    invoking this method has no effect.

    :   **Specified by:**: `close` in interface `SbrtFile`

---


### getOriginalName

```
public final java.lang.String getOriginalName()
```

:   Returns the name of this file.

    :   **Specified by:**: `getOriginalName` in interface `SbrtFile`
    :   **Returns:**: the name of this file.

---


### getCanonicalName

```
public final java.lang.String getCanonicalName()
```

:   Returns the canonical name of this file.

    :   **Specified by:**: `getCanonicalName` in interface `SbrtFile`
    :   **Returns:**: the canonical name of this file. **See Also:**: `File.getCanonicalPath()`

---


### isClosed

```
public boolean isClosed()
```

:   Indicates if this file has already been closed.

    :   **Specified by:**: `isClosed` in interface `SbrtFile`
    :   **Returns:**: `true` if this file is closed; `false` othewise.


---


|  |  |  |
| --- | --- | --- |
| **Overview** \| **Package** \| **Class** \| **Use** \| **Tree** \| **Deprecated** \| **Index** \| **Help** | |  |
| PREV CLASS   **NEXT CLASS** | **FRAMES**    **NO FRAMES**     **All Classes** |  |
| SUMMARY: NESTED \| FIELD \| CONSTR \| METHOD | DETAIL: FIELD \| CONSTR \| METHOD |  |


---
